# Unexpected Radiologic Findings for a Casting Type of Radiolucent Colorectal Foreign Body Composed of Polyurethane Foam

**DOI:** 10.1155/2016/4987105

**Published:** 2016-04-24

**Authors:** Emi Sanjo, Fumihiko Tamamoto, Shoichi Ogawa, Maiko Sano, Tetsunori Yoshimura, Miwako Nozaki

**Affiliations:** ^1^Department of Radiology, Tokyo Metropolitan Ohtsuka Hospital, 2-8-1 Minamiohtsuka, Toshima-ku, Tokyo 170-8476, Japan; ^2^Department of Radiology, Dokkyo Medical University Koshigaya Hospital, 2-1-50 Minamikoshigaya, Koshigaya-shi, Saitama 343-0845, Japan; ^3^Department of Surgery, Tokyo Metropolitan Ohtsuka Hospital, 2-8-1 Minamiohtsuka, Toshima-ku, Tokyo 170-8476, Japan

## Abstract

Radiologic diagnosis of colorectal foreign bodies is usually not very difficult, because inserted materials are often clearly visible on plain abdominal radiographs. However, when they are radiolucent, a plain abdominal radiograph has been reported to be useless. As radiolucent colorectal foreign bodies appear as radiolucent artificial contours or air-trapped materials in the pelvis, almost always the diagnosis itself can be made by careful evaluation of plain abdominal radiographs. We encountered a case of casting type of radiolucent colorectal foreign body formed from polyurethane foam. It presented us with unexpected radiologic findings and led to diagnostic difficulties.

## 1. Introduction

Colorectal foreign bodies (CRFs) are mainly a result of insertion of instruments into the anal canal and/or rectum, which cannot be removed. The most common category of CRFs is objects that are inserted voluntarily and for sexual stimulation [1, 2]. Usually, plain abdominal radiographs are enough for diagnosing CRFs, because most CRFs are clearly demonstrated as artificially shaped opacities in the pelvis. However, if they are radiolucent, plain abdominal radiographs are reported to be useless, because air-trapped sponges and hollow objects mimic loops of dilated bowels [3, 4]. Meanwhile, computed tomography (CT) is a useful modality for evaluating radiolucent CRFs, adverse abdominal events, such as colorectal perforations, anal lacerations, and/or hematomas. We report a case of a casting type of radiolucent CRF formed by polyurethane foam (PUF) with unexpected CT and radiographic findings that led to difficulties in radiologic diagnosis.

## 2. Case Report

**Figure 1:**
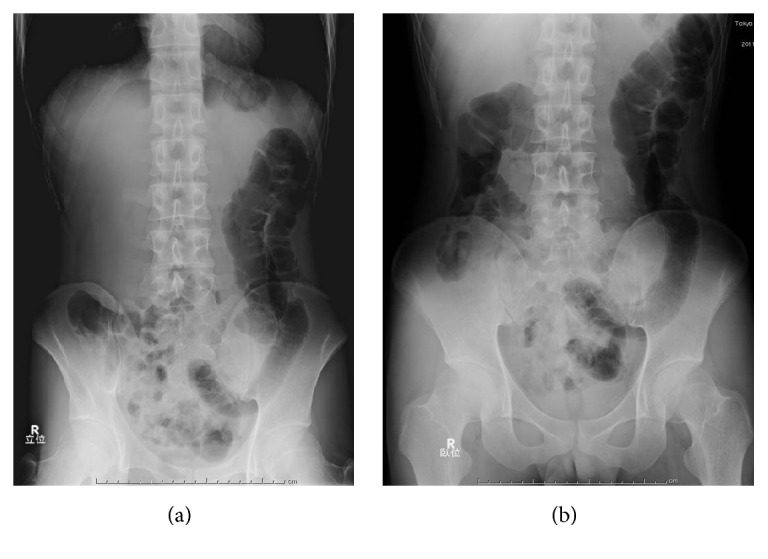
(a) Plain abdominal radiograph, upright position. (b) Plain abdominal radiograph, supine position. Images of gas in the left side of the colon in upright and supine positions are nearly identical.

**Figure 2:**
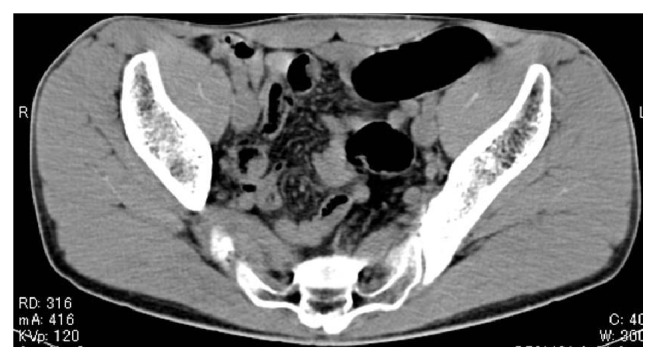
Initial computed tomography image. The image shows only normal gas distribution in the gastrointestinal tract; there is no indication of a foreign body.

**Figure 3:**
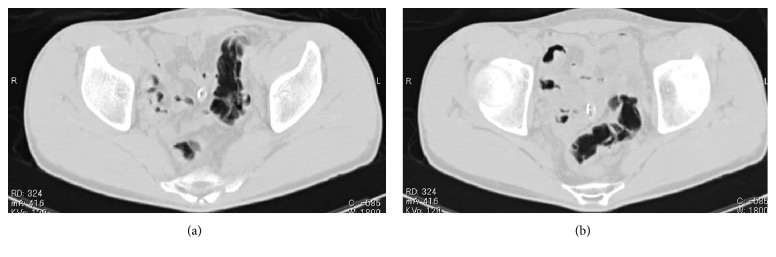
(a), (b) Repeat computed tomography image with an attenuation display. Reevaluation with an attenuation display revealing reticular strands from the distal transverse colon to the rectum probably corresponding to the foreign body.

**Figure 4:**
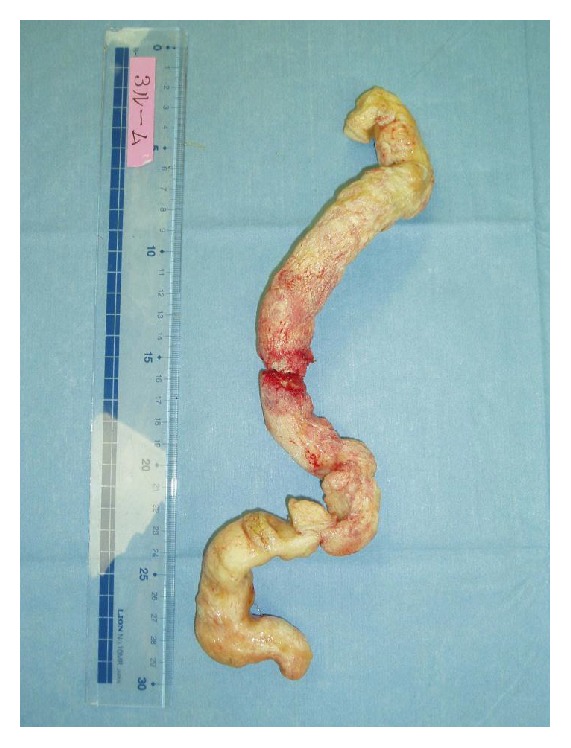
The foreign body removed from the gastrointestinal tract by surgery. Casting PUF was found to occupy the intestinal tract. The shape of the PUF fragment matched that of the section with gas detected on the left side of the colon in plain abdominal radiographs.

A 25-year-old man with no significant medical history presented with a rectal foreign body. According to the patient, it was PUF. He used to insert many different kinds of materials, but this was the first time that he could not retrieve it himself. Digital rectal palpation revealed a hard mass with a smooth surface in the anal canal. Laboratory findings and physical examinations were almost normal. A plain radiograph in upright and supine positions showed an increase of colorectal gas without bowel distension, but, unfortunately, an abnormal shadow that looked like a foreign body was not detected. The gas distribution in the rectum and left colon on both upright and supine positions was strangely almost equal (Figures 1(a) and 1(b)). On initial CT images for detecting the exact location of the foreign body and adverse abdominal events, perforation and intra-abdominal bleeding were not detected. Moreover, the foreign body itself and its exact location were not depicted contrary to our expectations (Figure 2). An endoscopy showed a yellowish mass with a sharply demarcated margin 7 cm from the anal ring, perhaps the lower edge of the foreign body. A physician promptly tried to retrieve it using alligator forceps, but the foreign body was fixed, brittle, easily crushed, and the strategy was not successful. Precise reevaluation of CT images taken with the air attenuation display setting revealed abnormal reticular strands from the distal transverse colon to the rectum, probably corresponding to the foreign body created by PUF (Figures 3(a) and 3(b)). We decided that it was impossible and dangerous to retrieve it per anus because it occupied a long segment on the left side of the transverse colon; therefore, operative removal was selected. The foreign body was removed through the incision in the sigmoid colon. The foreign body was composed of PUF and corresponded to the gas trapped on the left side of the colon on the plain radiograph (Figure 4). However, the PUF was brittle material and was easily crushed into many pieces by colonic peristalsis. Unexpectedly, it was distributed to the proximal portion of the colon, and we needed 4 other operations to remove the PUF completely.

## 3. Discussion

CRFs are objects like bottles and sexual toys that are inserted voluntarily for sexual stimulation [1, 2]. However, stool extraction using tools and insertions by patients with psychiatric disorders may also result in CRFs [5]. It can be difficult to elicit the exact history of a CRF because of patients' embarrassment. Prompt and exact diagnosis and treatment are essential because the CRFs may cause adverse abdominal events, for example, bowel perforation, anal laceration, and hematoma [1, 2]. Usually, a plain abdominal radiograph is used to establish CRFs but it can be useless if the CRFs are radiolucent [3, 4]. Glassware, wood sticks, corn cobs, and arms of vacuum cleaners are reported as radiolucent materials [6]. On the other hand, CT has been reported to be useful for detecting adverse abdominal events in case of radiolucent CRFs [4].

In our case, the abdominal radiograph only showed a gas-filled rectum and left colon and the presence of CRFs was not considered. At first, the abdominal CT also seemed to depict only a gas-filled rectum and colon. However, the gas distribution and the shape of gas-filled left colon on the upright and supine abdominal radiographs were almost identical. We called this the “fixed gas pattern,” which seemed to be an unexpected radiologic finding. CT images with air attenuation display revealed abnormal reticular strands from the distal transverse colon to the rectum, which seemed to correspond to CRFs formed by PUF; these were also unexpected findings.

PUF is an industrial product usually used for insulation in refrigerators and other equipment. The foam is made by the chemical reaction of two monomers (polyol and isocyanate; both viscous at room temperature), and when the two components are mixed, a foam almost 10–20 times the original volume is created in a few minutes. Finally, a hard, brittle sponge-like product is obtained. The patient inserted about 70 mL of the mixture into his rectum using a catheter-tip syringe. It expanded and occupied the rectum, left and distal portion of the transverse colon, forming a casting type of CRF.

The detection of the proximal edge of the CRF was also difficult because the PUF was so brittle that it was easily crushed into small pieces and mixed with stool on colonic peristalsis. Both plain radiographic and abdominal CT findings appeared to be almost normal, until CT with air attenuation display was employed. Considering these radiologic pitfalls, radiologists should keep in mind that casting type of radiolucent CRFs tends to present with almost normal radiologic findings and can mimic gas-filled bowels. The only radiologic way to correctly diagnose such CRFs is abdominal CT with an air attenuation display.

## 4. Conclusion

The unexpected radiologic findings, for example, fixed gas pattern, may be indicative of casting type of radiolucent colorectal foreign bodies. CT images with air attenuation display are essential for correct diagnosis in such cases.
